# Prototype of a Virtual Reality Simulator for Thyroidectomy: A Proof of Concept

**DOI:** 10.7759/cureus.92724

**Published:** 2025-09-19

**Authors:** Karl Eisentraeger, Eva Maria Dobrindt, Moritz Queisner, Christopher Remde, Igor M Sauer, Johann Pratschke, Martina Mogl, Frederike Butz, Charlotte Müller-Debus

**Affiliations:** 1 Department of Surgery, Campus Charité Mitte, Campus Virchow-Klinikum, Charité - Universitätsmedizin Berlin, Berlin, DEU

**Keywords:** dynamic model, endocrine surgery, immersive virtual reality, interactive medical image, medical education, medical simulation, simulation training, surgical training, thyroidectomy, virtual reality

## Abstract

The anatomical complexity of the thyroid region presents significant challenges in surgical training, particularly regarding the identification and preservation of the recurrent laryngeal nerve and parathyroid glands. We present a prototype of a virtual reality simulator designed to support thyroidectomy training by enabling the immersive, interactive exploration of CT-derived, deformable anatomical models in a photorealistic operating room environment. Structures not detectable in CT, such as nerves and glands, were manually integrated. The simulator was evaluated qualitatively by three surgeons using a structured questionnaire. Feedback indicated high usability, visual realism, and potential for improving anatomical recognition skills. Limitations include the absence of instrument interaction, haptic feedback, and full procedural simulation. This prototype demonstrates feasibility and outlines a clear development roadmap toward a high-fidelity, scalable training platform for endocrine surgery.

## Introduction

Thyroid surgery requires careful navigation around delicate structures within a confined anatomical space. Among the most critical structures are the recurrent laryngeal nerve and the parathyroid glands. Injury to the recurrent laryngeal nerve can lead to vocal cord palsy causing voice changes, hoarseness, and potential airway compromise, while inadvertent removal, devascularization, or injury of the parathyroid glands may cause permanent hypocalcemia requiring lifelong calcium supplementation [1-4]. Traditional surgical training encounters limitations: hands-on training in the operating room (OR) is restricted by safety imperatives, supervisor availability, and inconsistent, unpredictable case variations [5]. Moreover, due to the limited access to the surgical field, only the lead surgeon generally has an unobstructed view of all relevant structures, while the assistant's visibility is often restricted. To develop surgical proficiency, trainees must engage in repeated procedural practice to achieve technical accuracy and cultivate adaptive decision-making skills [6,7]. Translating knowledge from static educational materials and imaging to the dynamic surgical environment, where real-time decision-making is critical, poses a substantial perceptual and cognitive challenge. Anatomical recognition during operative procedures remains a capability requiring dedicated development. Physical anatomical training models present simplified anatomy with limited variation and authenticity [8,9]. Cadaveric training faces logistical challenges, ethical concerns, and high costs and lacks living tissue characteristics [10-15]. 

Virtual reality training using head-mounted displays is increasingly recognized as an effective and cost-efficient approach to address these gaps in surgical training, offering considerable potential to simulate complex scenarios and anatomical variations beyond the capabilities of traditional methods [8,16,17]. Virtual reality enables repeatable surgical training scenarios with high realism and interactivity, a potential that could also be used in thyroid surgery. We introduce a virtual reality-based thyroidectomy simulator prototype that integrates a photorealistic OR environment with CT-derived, deformable anatomical models. This system enables trainees to manipulate anatomical structures from authentic surgical viewpoints, facilitating the safe translation of theoretical knowledge into practical surgical skills while addressing the fundamental educational challenge of transitioning from static learning materials to dynamic operative reality. Built by an interdisciplinary team with thyroid surgeons, it pushes simulator realism toward full anatomical and procedural fidelity. The simulator is designed to facilitate the training of anatomical structure recognition for less experienced surgeons and students from the perspective of an expert surgeon. Here, we present the initial development of the prototype and the results of initial user feedback.

This report appeared briefly as a preprint (https://preprints.jmir.org/preprint/75409) due to an unintended submission and has been promptly removed.

## Technical report

The prototype was developed using Unity Pro (Version 6000.0.29, Unity Technologies, San Francisco, CA, USA) [18].

Simulator workflow

**Figure 1:**
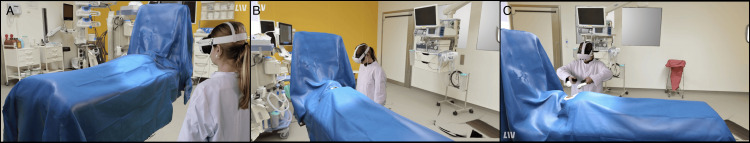
A colleague entering the simulation (A), regarding the surgical site (B), and interacting with the model (C) The virtual reality thyroidectomy training simulator shown here illustrates the user workflow: entering the photorealistic operating room environment (A), inspecting the exposed thyroid surgical field with surrounding critical structures (B), and interacting with the deformable anatomical model using controller-based ray‑guided manipulation, with deformations that can be retained or undone (C).

The simulator enables users to navigate freely within a virtual OR (Figure 1) [19]. The implemented interaction focuses on deformable tissue anatomy at the surgical site. Here, the users can prepare the neck muscles to gain sight of the thyroid, parathyroids, and major vessels. It is possible to move the thyroid gland in a plausible manner. All deformations can be undone with a single button press. The workflow was designed to ensure intuitive usability. This initial implementation simulates a stage of thyroid surgery in which the gland is fully exposed and mobilized. At this stage, the surgeon's primary objective is to identify and preserve critical structures, including the recurrent laryngeal nerve and the parathyroid glands, prior to resection.

Image data and segmentation

**Figure 2:**
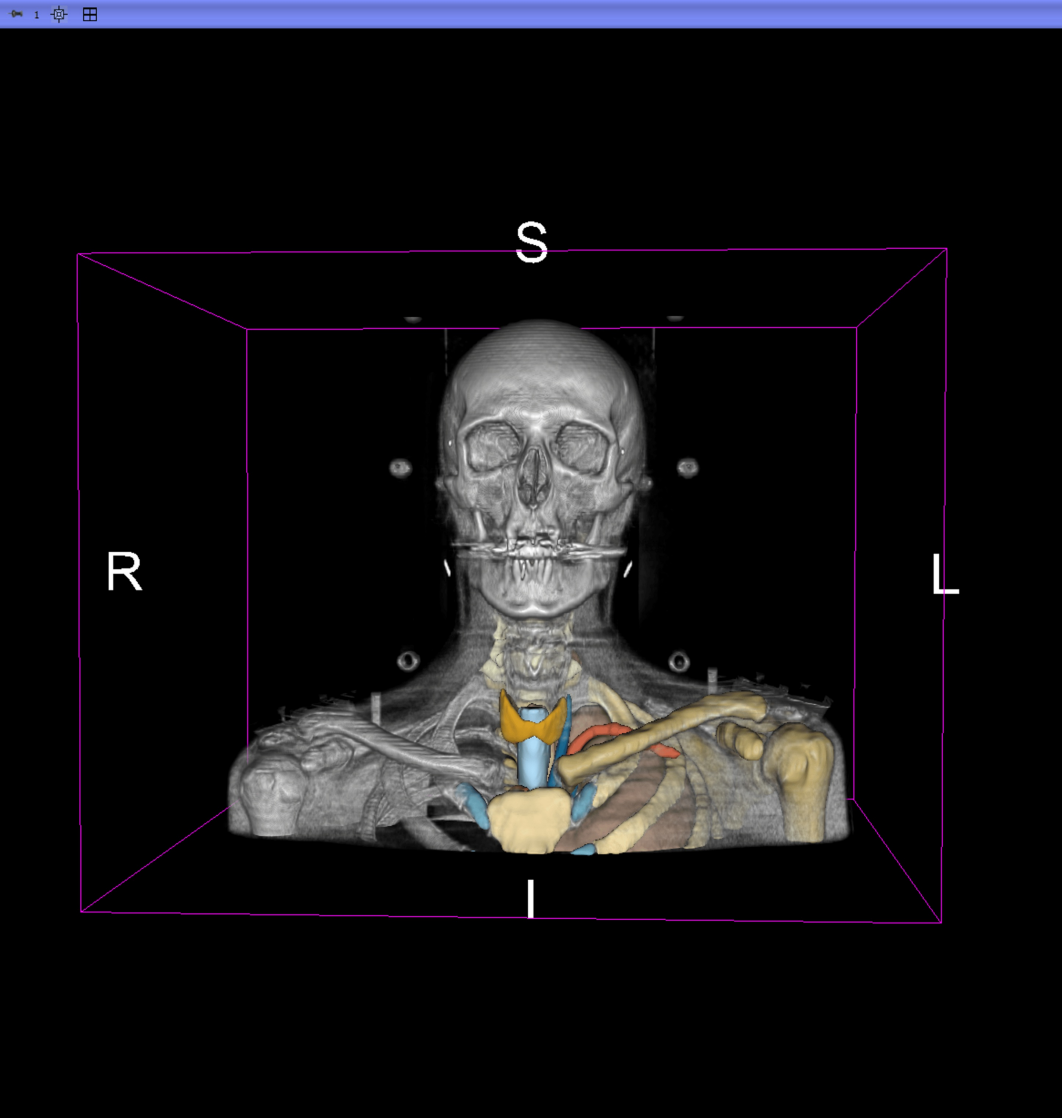
Segmentation with TotalSegmentator in 3D Slicer Volumetric rendering in 3D Slicer of an anonymized head‑and‑neck CT segmented with TotalSegmentator ("total," "headneck_bones," "headneck_muscles"); for visual clarity, only selected left‑sided segments and central midline structures are displayed, including the thyroid, to provide inputs for subsequent 3D model generation.

We used an anonymized publicly available CT scan as the basis for our anatomy model [20]. We imported the CT scan into 3D Slicer (Version 5.8.1, Brigham and Women's Hospital, Boston, MA, USA) [21] and performed segmentation using TotalSegmentator (University Hospital Basel, Basel, Switzerland) [22]. We used the total, headneck_bones, and headneck_muscles tasks [23] to segment the relevant anatomy (Figure 2).

3D model generation and organ deformation

**Figure 3:**
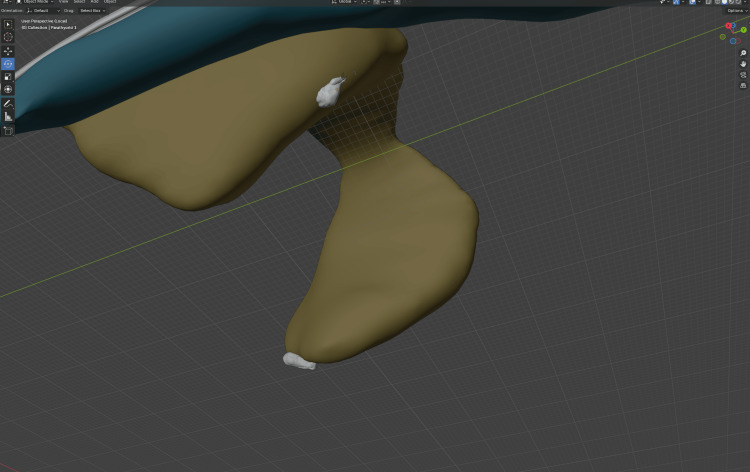
Manual insertion of the parathyroids in Blender Screenshot from Blender [24] illustrating the manual placement of the parathyroid gland meshes (white ovoids) relative to the thyroid model derived from CT-based segmentation. The parathyroids were created and positioned at typical posterior superior/inferior sites; the RLN was modeled and UV maps were assigned for later texturing (not shown). Base geometry originated from 3D Slicer/TotalSegmentator segmentations [21-23]. RLN: recurrent laryngeal nerve

**Figure 4:**
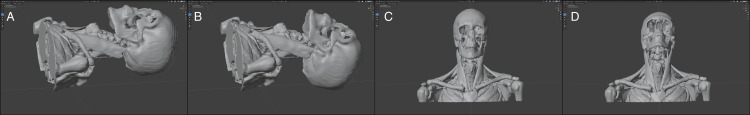
Tilting of the head implemented in Blender Multiple views (A-D) of manual neck hyperextension implemented in Blender [24] to reproduce the standard thyroidectomy position. Segment groups (skull and mandible, cervical spine, larynx-trachea complex, muscles) were rigidly transformed en bloc and then minimally adjusted to preserve continuity and prevent mesh intersections. The final pose replicates surgical hyperextension, improving the exposure of the thyroid compartment to match intraoperative anatomy.

**Figure 5:**
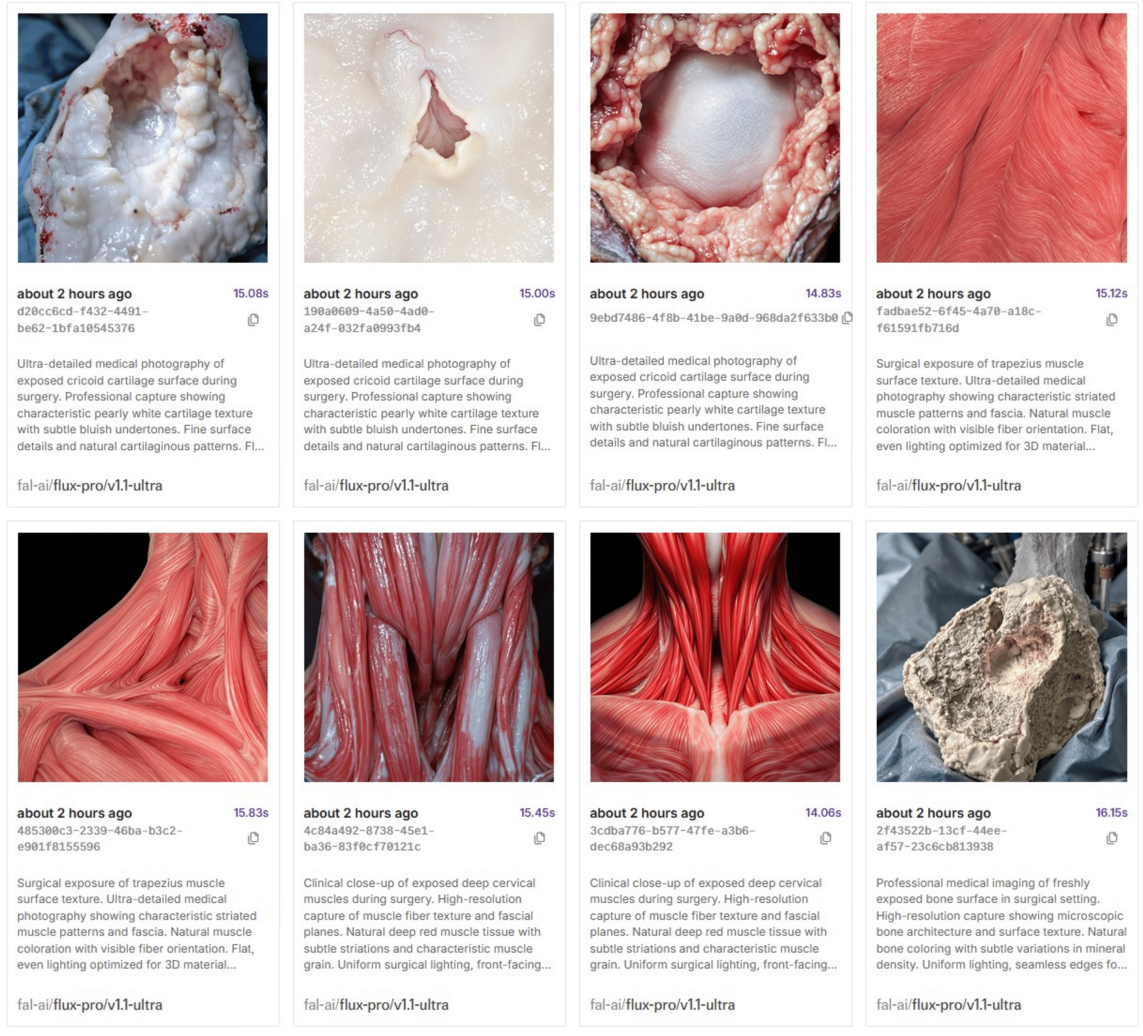
Preview of prompts and output generated by FLUX 1.1 Pro Ultra Examples of text prompts and corresponding AI‑generated texture previews produced with FLUX 1.1 Pro Ultra via the fal.ai API [25,26]. Prompts targeted organ‑specific textures (e.g., thyroid parenchyma, muscles, trachea, vessels) and were refined for color, microstructure, moisture sheen, and seamless tileability. Selected outputs were used to create texture sets for UV‑mapped meshes in Unity. All images are synthetic and used solely for texture authoring; no patient images are shown.

**Figure 6:**
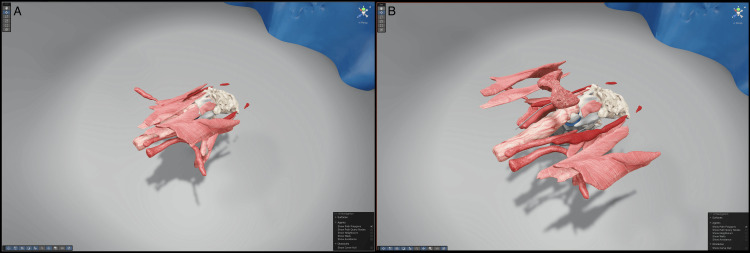
Final model isolated (A). The same model but exploded (B) Final virtual reality-ready anatomy model used in the simulator. (A) Integrated model in anatomical position. (B) Exploded view illustrating the constituent meshes and their spatial relationships, including the thyroid gland, muscles, trachea/laryngeal framework, major vessels, parathyroid glands, and recurrent laryngeal nerves.

The segmentations described under "Image data and segmentation" were exported as OBJ files to Blender (Version 3.0.0, Blender Foundation, Amsterdam, Netherlands) [24], a free and open-source 3D computer graphics tool. In Blender, we manually inserted the parathyroid glands and the recurrent laryngeal nerve and applied UV mapping to all OBJ files (Figure 3). To mirror the effect of hyperextension of the neck, as is customary during thyroid surgery, we manually adjusted the positioning and tilt of the segments (Figure 4). The enhanced OBJ files were then imported into Unity Pro (Version 6000.0.29, Unity Technologies, San Francisco, CA, USA) [18]. Textures were created using FLUX (FLUX 1.1 Pro Ultra, Black Forest Labs, Wilmington, DE, USA) through the fal.ai API (Fal, San Francisco, CA, USA) [25,26] (Figure 5 and Figure 6).

The following is an example prompt for texture generation: "Hyper-realistic thyroid gland surface texture as viewed intraoperatively. Slightly moist organic surface with Base color variations from deep crimson (vascular zones) to burgundy-red (parenchyma). Delicate fibrous capsule texture with faint follicle patterns. Branching arteriole networks (bright scarlet) and thrombosed veins (darker maroon). Semi-transparent areas revealing subsurface vasculature Subtle collagenous striations and adipose tissue inclusions Near-flat surface topography (0.1-0.3 mm variance) with minimal specularity Surgical field lighting creating subtle moisture sheen (0.2 roughness) Microscopic blood spots and capillary leakage details Seamless tileability for 3D model UV mapping PBR-ready format (Albedo/ORM/Normal maps) for Unity URP."

**Figure 7:**
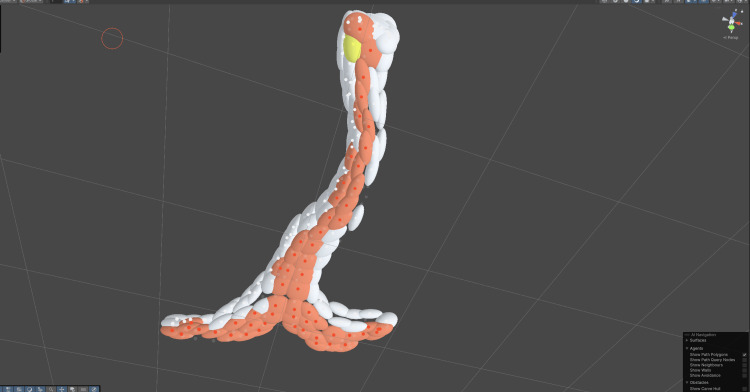
Selecting particles of the trachea in Unity using Obi Visualization of the soft‑body setup for the trachea in Unity Pro [18] using Obi Softbody [27]. The organ surface is discretized into simulation particles, grouped into clusters that define local stiffness and deformation behavior. The highlighted subset marks particles selected for constraint tuning and runtime interaction.

After exporting the OBJ files from Blender and importing them into Unity Pro, plausible tissue deformation was achieved using the real-time particle physics engine Obi Softbody (Obi Softbody Version 7.0, Virtual Method Studio, Madrid, Spain) [27]. Deformability was applied to select segmentations. Through Obi, we employed a particle-based method to discretize organs into clusters of particles. A shape-matching constraint system updates these particles' positions in real time to model soft-tissue deformation, with each cluster defining how its local region can deform through bending, stretching, and compression (Figure 7). A physics solver continuously computes the forces and constraints, adjusting particle positions to maintain the organ's overall shape. Simultaneously, plasticity parameters regulate permanent deformations to ensure realistic effects such as tissue stretch and recovery. These parameters were set with the aim of approximating real tissue behavior. Custom C# scripts were developed to enable virtual reality interaction with the deformable segmentations.

Virtual reality environment

**Figure 8:**
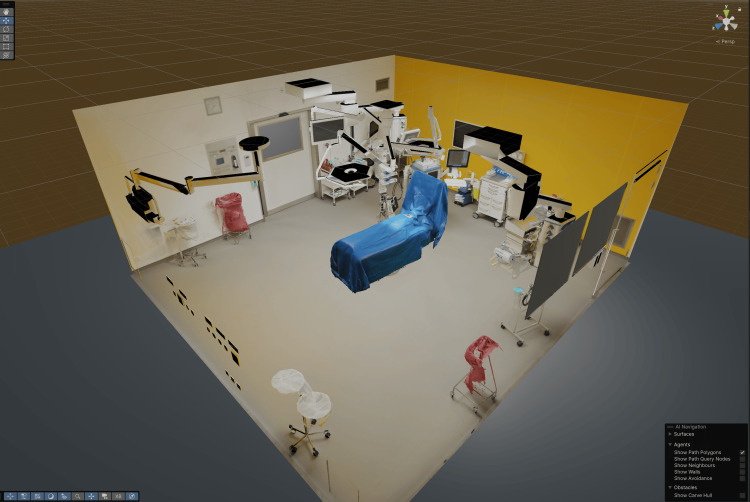
Operating room with patient model in Unity Unity-based virtual reality operating room [18] built from a photogrammetric reconstruction of the Charité - Universitätsmedizin Berlin operating room [19], integrating CT‑derived anatomy models, plus a photogrammetry‑derived patient, positioned and draped for thyroidectomy (hyperextended neck).

**Figure 9:**
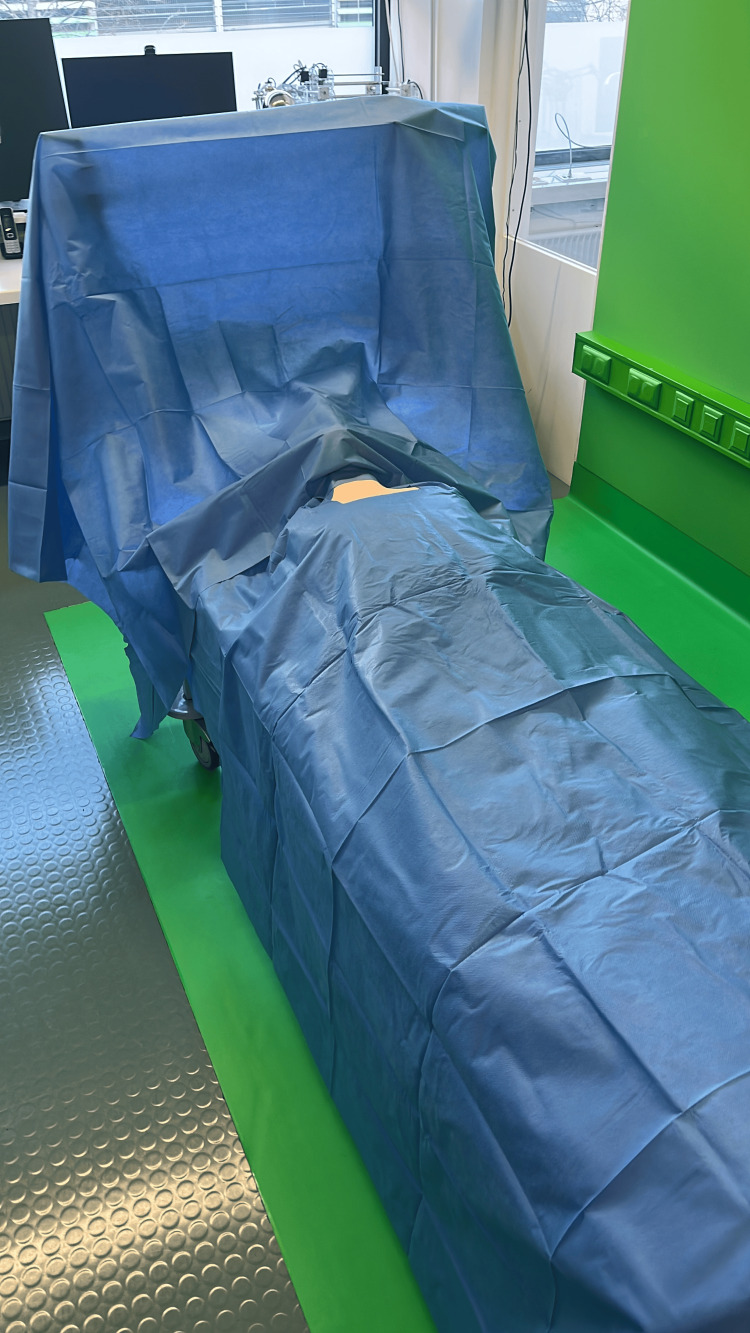
Draped mock patient Draped surgical training mannequin on an operating table in thyroid surgery position (neck hyperextended), photographed for the photogrammetry dataset used to build the patient model.

**Figure 10:**
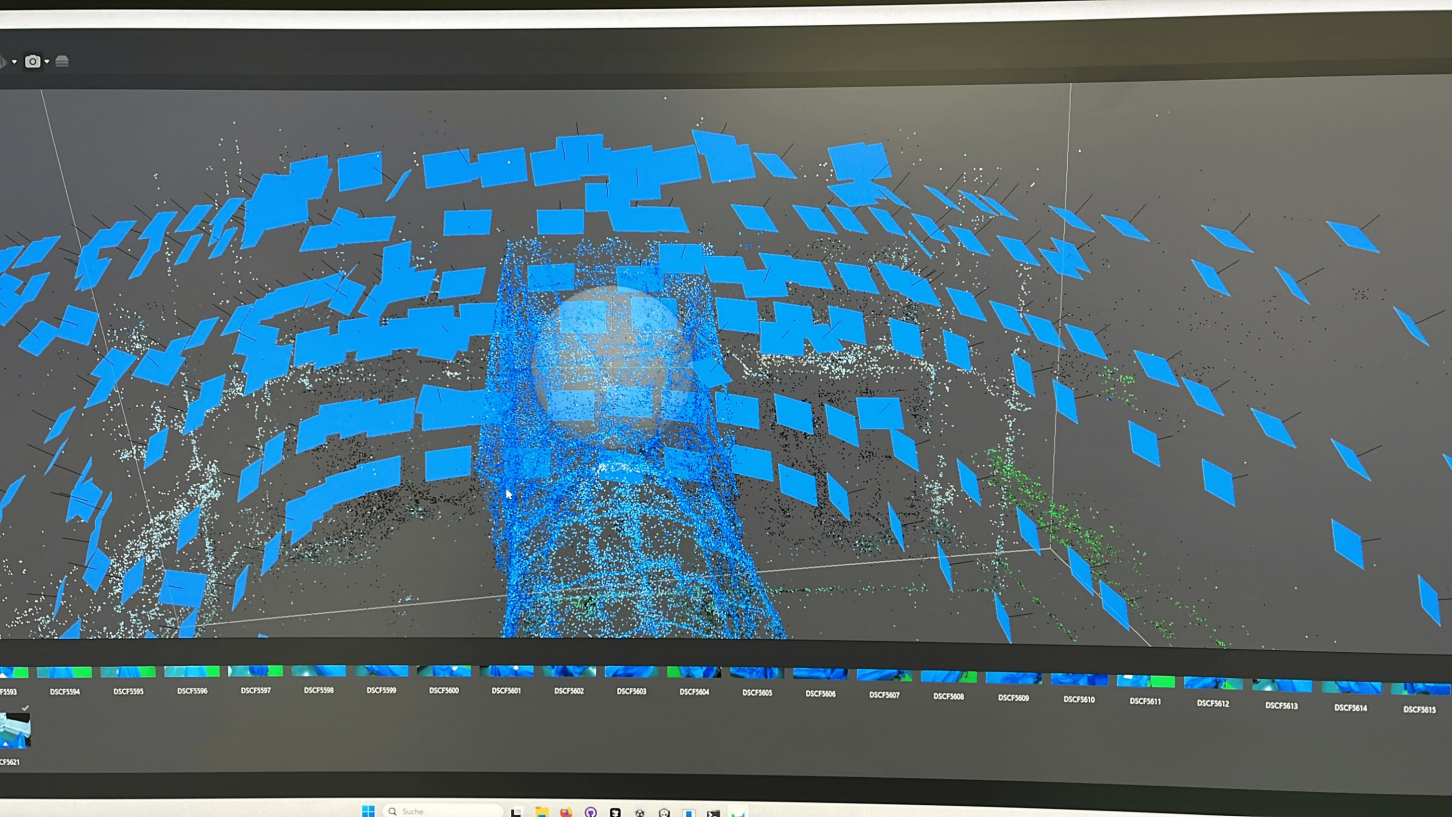
Generating a 3D model in Agisoft Photogrammetry in Agisoft Metashape: >300 uniformly lit, fixed‑exposure, tripod‑mounted photos (Fuji X-T4; ISO 160, f/8, 1/4 s) of a draped surgical mannequin were aligned into a dense point cloud and then meshed and texture‑mapped to create the simulator's patient model.

The environment recreates a fully equipped modern OR, featuring a centrally positioned operating table with a patient prepared for thyroid surgery (Figure 8). The scene is based on earlier work on a photogrammetric reconstruction of an OR at Charité - Universitätsmedizin Berlin, Campus Charité Mitte, in Berlin, Germany [19]. The patient model was newly created through high-resolution photogrammetry, utilizing a Fuji X-T4 camera (FUJIFILM Corporation, Tokyo, Japan) [28] mounted on a tripod with controlled settings (ISO 160, f/8 aperture, 1/4 second shutter speed). Under consistent light-emitting diode (LED) lighting conditions, we captured >300 photographs of a surgical training mannequin positioned on an operating table, with surgical positioning and draping (Figure 9). The photographic dataset was processed using Agisoft Metashape software (Agisoft Metashape Standard, Version 2.1.0 build 17532 (64 bit), Agisoft LLC, St. Petersburg, Russia) [29] to generate a detailed point cloud, which was then converted into a textured 3D model (Figure 10). The final model represents a thyroid surgery setup, featuring the characteristic hyperextended neck position and standard surgical draping protocols.

User interaction and interface

We developed the interaction system on top of the XR Interaction Toolkit (XRI Toolkit 3.0.7, Unity Technologies, San Francisco, CA, USA) [30]. Navigation utilizes the standard XRI implementation for passive directional movement controlled by the left controller's joystick and snap turning via the right controller's joystick. The remaining interactions are customized: when the trigger button of the right controller is pressed, the first particle from the anatomical models that collides with the controller's emitted visible ray will be subject to force. The applied force vector directly corresponds to the controller's positional and rotational state, maintaining consistent directional alignment. Upon release of the trigger, the deformation persists. The system maintains a sequential history of all deformations in memory. By pressing a dedicated button on the right controller, users can undo the last manipulation and revert the tissues to their previous state. This user interaction design facilitates the comprehensive exploration of the virtual model. In Video 1, the use of the prototype is shown from a first-person perspective.

**Video 1:** Virtual reality thyroidectomy surgery simulator prototype First‑person demonstration of the virtual reality thyroidectomy training prototype: the user freely navigates a virtual operating room, inspects the draped patient, and manipulates deformable neck tissues to expose and mobilize the thyroid while visualizing the parathyroids, recurrent laryngeal nerve, and major vessels; deformations persist and can be undone with a single controller button.

Hardware used for testing

The simulation was tested on a Quest 3 tethered via a USB-C 3.1 cable to a high-performance workstation. This workstation was equipped with an NVIDIA RTX 3090 graphics card (24 GB GDDR6X, Nvidia Corporation, Santa Clara, CA, USA), 64 GB of DDR RAM, and an AMD Ryzen 9 3900 12-core 3.80 GHz processor (Advanced Micro Devices, Inc., Santa Clara, CA, USA). In the scene, there were approximately six million vertices, and the program was running at about 70 frames per second.

Qualitative assessment

Three surgeons with varying levels of experience participated in testing the simulator after a brief training session on virtual reality controller navigation and interaction. The participants included a resident surgeon (one year of surgical experience), a board-certified visceral surgeon (2.5 years of surgical experience), and a consultant surgeon specialized in endocrine surgery (10 years of surgical experience); none had prior virtual reality experience. A custom qualitative questionnaire was developed to assess the following domains: general impressions, immersion and realism, anatomical accuracy, tissue behavior and haptics, interaction and controls, learning and training effectiveness, user interface and workflow, additional features and future enhancements, and overall evaluation and feedback (Appendix 1 (German) and Appendix 2 (English)). Complete questionnaire responses are presented in tabular format in Appendix 3.

The surgeons agreed that the prototype offered a realistic experience capable of improving effective operating practice and learning. The simulation was perceived as providing an immediately convincing OR environment. Although it did not replicate authentic surgical maneuvers, participants reported that interactions became intuitive and accessible after a brief familiarization period. The prototype was considered particularly valuable for teaching anatomical landmarks, especially nerve tracts and the locations of the parathyroid glands. Participants suggested multiple enhancements to improve simulator fidelity, including more realistic organ responses, integration of standard surgical instruments, addition of comprehensive tissue layers, implementation of an authentic OR sound atmosphere, and incorporation of haptic feedback for tactile sensation.

Ethical considerations

This study focused on developing and qualitatively evaluating an educational virtual reality thyroidectomy training simulator prototype. Anatomical models were based on anonymized public CT data. Written informed consent was obtained from the three testing surgeons. A surgical training model was used for photogrammetry to create the patient setup. No procedures were performed on humans or animals, and no sensitive data were collected. According to institutional guidelines, this type of educational technology research does not require formal institutional review board approval. All activities complied with the Declaration of Helsinki and institutional policies. Surgeon feedback was documented and analyzed with consent.

## Discussion

This technical report presents the development of a prototype virtual reality thyroidectomy training simulator that integrates CT-derived dynamic anatomical models within a photorealistic OR environment. Our goal is to describe initial iteration development approaches and assess their feasibility.

A preliminary qualitative assessment of the thyroid surgery simulator prototype offers insights into its current strengths and limitations. Feedback from expert surgeons provides a clear direction for future development and situates the simulator within the broader context of surgical training and its established challenges.

Strengths and educational potential

According to the surgeons, the simulator's primary strength lies in its ability to deliver a highly immersive and realistic training environment. The combination of a photorealistic OR with anatomically precise models derived from CT scans successfully fosters a strong sense of presence and engagement. Furthermore, the simulator's intuitive interaction design, which required minimal instruction, was identified as a significant advantage. This high level of usability reduces the barrier to entry, allowing users to focus on the task rather than on manipulating the system.

Comparison with existing thyroidectomy trainers

Compared with current thyroidectomy trainers, largely physical benchtop models, and biological wet labs, our virtual reality prototype provides an immersive OR built from CT‑derived patient anatomy with real‑time deformable interaction. The transoral endoscopic thyroidectomy vestibular approach (TOETVA) thermoplastic box trainer demonstrates good face/content validity but has fixed anatomy and is designed to simulate the transoral endoscopic approach, not open‑field dissection [31]. Animal and cadaveric models permit tissue handling yet are anatomically mismatched and resource‑intensive [8], and intraoperative augmented reality tools support experts rather than train novices [8]. A recent Apple Vision Pro virtual reality trainer targets intraoperative neuromonitoring with hand tracking, artificial intelligence (AI) assistance, and performance analytics. However, it only focuses on neuromonitoring electrode placement rather than open surgical exposure. In addition, it employs stylized, non‑photogrammetric visuals with predominantly binary, trigger‑driven interactions, distinct from our photogrammetry‑based OR and CT‑based anatomy [32]. Reviews and a contemporary trial registration underscore the scarcity of immersive virtual reality thyroidectomy trainers [8,33]. Although our current prototype lacks automated metrics and case variability, its patient‑data foundation can evolve toward proficiency‑based curricula, aligning with meta‑analytic evidence that digital/virtual reality training matches or exceeds traditional labs and that simulator performance predicts OR performance [34,35].

Performance and scalability challenges

The current implementation achieves a frame rate of 70 FPS while rendering approximately six million vertices on high-end hardware. However, this performance benchmark reveals critical scalability concerns. The particle-based deformation system introduces substantial memory overhead, which increases exponentially with anatomical complexity. As we expand the model to incorporate multiple tissue layers, fascial planes, and vascular networks, maintaining real-time performance across diverse hardware configurations will present computational challenges requiring optimization strategies.

Anatomical modeling limitations and validation framework

CT scan resolution constraints significantly limit model fidelity, while manual insertion of non-visible structures such as the recurrent laryngeal nerve and parathyroid glands presents substantial challenges in achieving anatomically accurate positioning with reproducible precision. The current reliance on expert feedback for the validation of training scenarios, though valuable, lacks the standardization and objectivity required for systematic model improvement. Future development should prioritize advanced image processing techniques, potentially incorporating multi-modal imaging fusion and machine learning algorithms, to automatically predict and position structures not clearly visible in standard CT imaging, thereby establishing a more robust and scalable validation framework.

Interaction and haptics

The current ray-casting interaction system, though functional for basic manipulation, critically lacks the haptic feedback essential for authentic surgical skill development. Without force resistance, texture sensation, and proprioceptive cues, a significant disconnect exists between virtual manipulation and real surgical experience. Integrating comprehensive haptic systems, precision hand-tracking, and intuitive gesture recognition presents substantial technical challenges requiring breakthrough advances in hardware integration and real-time software optimization to bridge the gap between current virtual capabilities and the tactile complexity of surgical practice.

Physics-based material modeling evolution

The current approach to tissue deformation, while visually plausible, relies on generalized parameters that fail to accurately capture the distinct mechanical properties of individual anatomical structures. Advancing toward rigorously data-driven deformation models represents a significant challenge and a key opportunity. This evolution would require assigning tissue-specific parameterization derived from comprehensive mechanical testing data to each anatomical component. Such an approach would incorporate precise biomechanical material properties that govern tissue deformation under applied stress, including Young's modulus, Poisson's ratio, viscoelastic coefficients, and anisotropic behavior patterns specific to the thyroid parenchyma, muscle tissue, fascial layers, and vascular structures.

Automated workflows for non-technical users

The current CT-to-deformable-model pipeline creates significant bottlenecks that limit rapid iteration and efficient content creation workflows. Asset optimization is primarily a manual process that requires specialized technical knowledge, creating barriers for medical educators and content creators who lack this expertise.

Future development should focus on implementing fully automated pipelines with built-in quality control systems. This automation would enable users without technical backgrounds to generate anatomically accurate models through user-friendly interfaces while maintaining the rigorous standards required for medical education applications.

Limitations

Despite its promise, the prototype has clear limitations that currently hinder its integration into clinical training programs. The primary limitation is that this virtual reality simulator still represents an initial and early-stage prototype. In this initial version, the surrounding tissue, the handling of surgical instruments, the dissection of tissue, and the correct anatomical mobility could not be accurately modeled. These deficiencies, while expected in an early prototype, must be addressed to elevate the prototype from a basic anatomical demonstrator to a comprehensive surgical skills trainer.

## Conclusions

This proof of concept demonstrates the feasibility of virtual reality-based thyroidectomy training through CT-derived deformable anatomy within a photorealistic operating environment. Qualitative assessment by three surgeons confirmed high usability and anatomical recognition value while identifying specific limitations. This feedback directly establishes development priorities, multilayered tissue implementation, deformation physics optimization, and instrument integration. Though results are preliminary given the small sample, the prototype addresses key training constraints including limited OR access and cadaveric availability. Future iterations will expand case diversity and pursue the quantitative validation of educational effectiveness, working toward a comprehensive platform that standardizes thyroidectomy training and enhances patient safety by allowing surgeons to gain experience before entering the OR.

## Appendices

Appendix 1: Fragebogen für Chirurg*innen zur Evaluation des VR-Schilddrüsen Operationssimulators 

Allgemeiner Eindruck

Was war Ihr erster Eindruck, als Sie die Simulation gestartet haben?

Immersion und Realitätsnähe

Wie beurteilen Sie das Gefühl des „Eingetauchtseins“ in die virtuelle Umgebung? (Bitte beschreiben Sie, ob Sie sich in die OP-Situation versetzt fühlten oder ob es Barrieren gab.) 

Welche Aspekte (z.B. visuelle Darstellung, Geräusche, Interaktion) verstärken Ihrer Meinung nach die Immersion, und was könnte verbessert werden? 

Anatomische Plausibilität

Wie authentisch wirkt die Darstellung der anatomischen Strukturen, insbesondere der Schilddrüse, Blutgefäße, umliegenden Gewebe etc.? (Bitte beschreiben Sie, ob die Gewebe realistisch angeordnet und erkennbar sind.)

In welchen Bereichen wünschen Sie sich eine noch höhere anatomische Detailgenauigkeit oder eine bessere Darstellung? 

Deformation und Haptik

Wie realistisch empfanden Sie die Verformung des Gewebes (z.B. Elastizität der Schilddrüse, Verschieblichkeit der umliegenden Strukturen)? (Gibt es Bereiche, in denen die Deformation zu starr oder zu weich wirkt?)

Welche Verbesserungen würden Ihrer Meinung nach die Plausibilität der Gewebe-Interaktion weiter erhöhen? 

Bedienung und Steuerung

Empfinden Sie die aktuellen Interaktionsmöglichkeiten (z.B. Greifen, Verschieben) als intuitiv? (Falls nein: Was sind die Hauptschwierigkeiten?)

Welche weiteren Interaktionsformen würden Sie sich wünschen (z.B. zusätzliche Instrumente, Ultraschall-Integration, kraftbasiertes Feedback etc.)?

Lern- und Übungseffekt

Welche konkreten Lernziele würden Sie beim Einsatz dieses Simulators verfolgen (z.B. Erlernen anatomischer Landmarken, Umgang mit Instrumenten, Abfolge einer OP)? 

Wie gut unterstützt der Simulator Sie bisher bei der Erreichung dieser Lernziele, und welche inhaltlichen Schwerpunkte sollten ggf. erweitert werden? 

Benutzeroberfläche und Workflow

War die Navigation in der Simulation (Menüführung, Einstellungen etc.) klar verständlich? (Bitte nennen Sie konkrete Vorschläge für Verbesserungen oder Ergänzungen.)

Inwieweit fügt sich die Bedienung des Simulators in Ihren üblichen klinischen Workflow ein (z.B. Zeitaufwand, Einarbeitung)? 

Wünsche und Ausblick

Welche zusätzlichen Funktionen oder Szenarien würden Sie sich für künftige Versionen wünschen (z.B. komplexere Pathologien, mehr OP-Schritte)?

Welche Realismus-Aspekte sind für Sie persönlich besonders entscheidend, um sich langfristig mit dem Simulator beschäftigen zu wollen? 

Gesamtbewertung und Abschluss

Was hat Ihnen an der Simulation besonders gut gefallen, und was sollte Ihrer Meinung nach dringend überarbeitet werden? Möchten Sie noch etwas hinzufügen oder haben Sie weitere Ideen / Anmerkungen? 

Appendix 2: Questionnaire for surgeons on the evaluation of the virtual reality thyroid surgery simulator

General Impression

What was your first impression when you started the simulation?

Immersion and Realism

How do you assess the feeling of "immersion" in the virtual environment? (Please describe whether you felt transported into the surgical situation or if there were barriers.)

Which aspects (e.g., visual representation, sounds, interaction) in your opinion enhance immersion, and what could be improved?

Anatomical Plausibility

How authentic does the representation of anatomical structures appear, particularly the thyroid, blood vessels, surrounding tissues, etc.? (Please describe whether the tissues are realistically arranged and recognizable.)

In which areas would you like to see even higher anatomical detail accuracy or better representation?

Deformation and Haptics

How realistic did you find the tissue deformation (e.g., elasticity of the thyroid, mobility of surrounding structures)? (Are there areas where the deformation seems too rigid or too soft?)

What improvements would, in your opinion, further increase the plausibility of tissue interaction?

Operation and Control

Do you find the current interaction possibilities (e.g., grasping, moving) intuitive? (If no, what are the main difficulties?)

What additional forms of interaction would you like to see (e.g., additional instruments, ultrasound integration, force-based feedback, etc.)?

Learning and Training Effect

What specific learning objectives would you pursue when using this simulator (e.g., learning anatomical landmarks, handling instruments, sequence of an operation)?

How well does the simulator currently support you in achieving these learning objectives, and which content areas should potentially be expanded?

User Interface and Workflow

Was navigation in the simulation (menu guidance, settings, etc.) clearly understandable? (Please provide specific suggestions for improvements or additions.)

To what extent does operating the simulator fit into your usual clinical workflow (e.g., time investment, familiarization)?

Wishes and Outlook

What additional functions or scenarios would you like to see in future versions (e.g., more complex pathologies, more surgical steps)?

Which realism aspects are particularly crucial for you personally to want to engage with the simulator long-term?

Overall Assessment and Conclusion

What did you particularly like about the simulation, and what do you think should be urgently revised?

Would you like to add anything else or do you have further ideas/comments?

Appendix 3

**Table 1:** Qualitative feedback summary from VR thyroidectomy simulator evaluation Three surgeons with varying experience levels (consultant endocrine surgeon, board-certified visceral surgeon, and resident surgeon) evaluated the VR thyroidectomy simulator prototype using a structured 10-question assessment. Responses were collected after a brief familiarization session and cover domains including immersion, anatomical accuracy, tissue behavior, interaction design, learning effectiveness, and future development priorities. All participants provided written informed consent for the documentation and analysis of their feedback. VR: virtual reality; OR: operating room; EKG: electrocardiogram

Question	Consultant surgeon specialized in endocrine surgery	Board-certified visceral surgeon	Resident surgeon
(1) What was your first impression when you started the simulation?	Impressively realistic, the OR is very realistic and you immediately find yourself in your position as a surgeon, want to immediately approach the table.	Very good impression, very realistic work environment, OR site appears very realistic at first glance.	The OR environment seemed very realistic, as did the position of the "patient" on the OR table. I was able to put myself into an OR situation.
(2) How do you assess the feeling of "immersion" in the virtual environment? Which aspects (e.g., visual representation, sounds, interaction) enhance immersion in your opinion, and what could be improved?	Immediately immersed in the situation, the OR environment is very real as the perspective realistically changes with viewing direction. The floating anatomy model at the door reduced immersion initially as it cannot be real in that position. Still felt well into the OR situation. Could be supported by OR sounds - EKG beeping and ventilator sounds. The absence of other personnel (anesthesiologist, nursing staff) feels somewhat "strange" and unusual for a running operation. When approaching the OR table, the "aloneness" fades and you're in the real situation at the OR table. Interaction with controls doesn't match natural movement patterns when moving in space using the joystick for approach, but this can be mentally blocked. You want to intuitively approach the OR table immediately. Could be fixed by starting directly at the OR table. At the OR table, the visual representation of the incision edge seems somewhat strange, but the view is immediately directed to the site. Would like it larger/zoomed since thyroid surgery also works with magnifying glasses. Want to get closer to the model.	Found it convincing "immersion." Almost forgot I was actually standing in the office. Somewhat strange and perhaps not optimal was that the OR table was initially much too high or I had a very large distance to the OR table; approaching the OR table correctly was not quite easy at first. Think this has much to do with "handling" that must be learned first. The "problems" could also be well resolved.	Could immerse myself very well in the OR situation. A few things were less intuitive: 1. movement in space (forward/backward through controller movement and not through own movement in space). Additionally, you can "walk through the table." 2. The height of the OR table could not be changed within the model, so it was sometimes difficult to get a view from above on the site. The virtual OR environment that resembles our ORs definitely enhances immersion. Sounds could also do this, though they shouldn't be disturbing noises (which unfortunately often occur in reality in the OR). Meaningful sounds would be e.g. the neuromonitoring signal when detecting the nerve.
(3) How authentic does the representation of anatomical structures appear, particularly the thyroid, blood vessels, surrounding tissue, etc.? In which areas would you like higher anatomical detail accuracy or better representation?	The thyroid is very authentically captured from its surface. The large blood vessels as well, but the position of the N. Vagus is too superficial, as it doesn't actually lie on the large neck vessels but is hidden deep between vein and artery and only becomes visible after spreading the two structures. The muscle surface is also realistic. The parathyroid glands are very difficult to find - which corresponds to reality. Actually, you would expect the position of the upper ones more centrally and toward the upper pole, the lower ones correspond somewhat to the expected localization. What's unrealistic from the surgeon's perspective is that they attach to the thyroid, which is rather rare in surgery - but corresponds to the situation where you "weren't paying attention" and didn't prepare strictly capsule-close and they then attach to the capsule and in the worst case are removed unnoticed. Since no fat tissue is integrated in this model yet, everything looks very "naked," which often doesn't correspond to reality in the OR and actually makes identification of parathyroid glands more difficult. In real surgery, you would prepare the thyroid capsule-close and thereby reach between thyroid and parathyroid, whereby these come to lie "underneath" (anatomically dorsal to the thyroid) in the fat tissue. The NLR is very fine and thus very realistic in size, perhaps somewhat paler/lighter in nature and somewhat too deep (footward) in feeling.	The thyroid appeared very realistic in its optics. The position of the parathyroid glands is very variable, but the positions could be somewhat optimized (for example, I would place the lower parathyroid glands more at the lower edge than on the thyroid front). The N. Laryngeus is somewhat too difficult to recognize, I wasn't sure if it's represented in the lower area on the trachea. It runs only laterally to the trachea. The nerve course should run close to the thyroid base before entering the larynx - approximately where the thyroid is fixed to the trachea in the simulation. The N. Vagus should run somewhat deeper between A. Carotis and V. Jugularis. As a color, I would rather take yellow and not gray-green. The musculature and trachea I find already quite well represented. You could perhaps also represent the esophagus.	Musculature: M. Sternocleidomastoideus - very authentic. Straight neck musculature - also appears largely authentic. What's missing overall compared to reality is the connective tissue ("filling tissue") between the structures, which makes it appear less realistic at first glance. Thyroid: The surface appears realistic, as does the position and shape. Blood vessels: realistic position and courses - thus simple identification possible. Parathyroid glands: The coloring seems somewhat too dark and the position particularly with the two caudal parathyroid glands somewhat too far ventral (but such variants certainly exist). N. Vagus: seems not authentic in color and more like a green/blue rubber band. Regarding position, it would probably be found somewhat deeper between Carotis and V. Jugularis. N. Laryngeus recurrens: this is very difficult for me to distinguish from the tracheal surface in color, which certainly corresponds to reality to a certain degree. Nevertheless, I think the branching in relation to the larynx occurs somewhat too deep. Here I would wish for somewhat more detail accuracy.
(4) How realistic did you find the deformation of tissue (e.g., elasticity of thyroid, displaceability of surrounding structures)? In which areas does the deformation appear too rigid or too soft? What improvements would increase the plausibility of tissue interaction?	The thyroid was super flexible, which is too soft compared to reality, as you could luxate it too easily. The fixation point would ideally be at the insertion of the N. Laryngeus recurrens. Currently, the nerve lies free - as it actually is only in the final step of the operation.	The mobility is somewhat more pronounced than in reality. The mobility of the thyroid is only possible to this extent after complete mobilization of the thyroid. The musculature and thyroid are not quite so "chewing gum-like." Overall, I find the mobility really great.	The displaceability of the muscles I found largely realistic. The thyroid itself was very mobile and appeared very soft. Usually, it is first mobile after preparation and also somewhat stiffer in tissue consistency. Unrealistic is that the thyroid can be pulled upward through the musculature. So only the thyroid moves and deforms and the overlying layers (musculature) remain unchanged. I didn't try to deform the trachea itself. It should also be quite rigid.
(5) Do you find the current interaction possibilities (e.g., grasping, displacing) intuitive? What are the main difficulties? What additional interaction forms would you wish for (e.g., additional instruments, ultrasound integration, force-based feedback, etc.)?	Essentially yes - it doesn't correspond to reality, but it's intuitive. If you go into detail, for example the following situation is somewhat "strange": you point at the straight neck muscle of the opposite side and "push" it away to open the site - in reality, such "maneuvers" are performed by inserting a hook, grasping and a "pull" movement. But this is due to the model and doesn't significantly disturb the OR experience.	I basically found the hand movement intuitive. But I had some difficulties with the pointer's targeting accuracy: sometimes I wasn't clear beforehand which tissue I was moving, as I couldn't well recognize the endpoint of the pointer. Force-based feedback would naturally be great - since you can only pull the thyroid with a certain amount of force, otherwise the nerve is endangered. Additionally, the integration of instruments (scissors, tweezers, Overholt, bipolar) would naturally be ideal, particularly also the integration of the neuromonitoring probe. When the nerve is detected with the probe, a positive signal could sound.	1. Movement in space through movement of the small joystick on the controller I find initially not intuitive, particularly when head movements/rotations occur "in reality" (must). 2. Grasping/displacing with the controller I find moderately intuitive. Initially, you must get used to the process and train it briefly. Then it's quite intuitive. However, I found the grasping/displacing possibility not particularly precise. I needed several attempts multiple times to exactly displace and grasp the tissue I wanted to grasp. 3. Further desired interaction forms: Neuromonitoring probe; tweezers with which you can grasp; hooks with which you can e.g. fix the musculature to hold it aside. Force-based feedback would naturally also be very desirable.
(6) What concrete learning goals would you pursue when using this simulator (e.g., learning anatomical landmarks, handling instruments, sequence of an operation)? How well does the simulator support you in achieving these learning goals, and which content focuses should be expanded?	Measured against the current state of the model: Learning anatomical landmarks, sequence of a standard operation. For learning anatomical landmarks, it's certainly already well advanced. For the level of students, it would be completely sufficient at the current time, as positional relationships between NLR, NV, thyroid, neck vessels and parathyroid glands are already very well illustrated, these learning goals are already achieved. For physicians in training, the addition of lymph and fat tissue would be desirable, as this creates even better reality proximity and increases difficulty, but the visualization of critical OR steps before the real OR is also an adequate learning goal for young physicians. For the sequence of the standard operation, the following must be present: the vessel strands at upper and lower pole and the central artery, as these must be consciously severed. Perhaps also the embedding fat tissue from which the organ is gradually shelled out. For this, instruments could naturally serve, but in the first step of a simple model, the identification of the resection boundary using the pointer would be a first good learning effect/learning goal.	Recognizing anatomical structures and being able to trace the course of nerves and the position (variability) of parathyroid glands in detail. Maintaining the OR steps in the correct sequence. Using instruments and the neuromonitoring probe correctly. Moving the thyroid in the correct extent/learning not to pull too strongly. Currently, you can already learn much about anatomy and find your way around the OR site. You can already experience where the nerves and parathyroid glands are located in relation to the thyroid. For me, a good content focus would be the positional variability of parathyroid glands (with the challenge of finding parathyroid glands) and finding the N. Laryngeus recurrens.	For the simulator in its current form, the concrete learning goals would be learning anatomical landmarks and their relation to each other. Goal for further development/a more complex simulator would be performing/sequence of a thyroid operation and training the identification of critical structures like the N. Laryngeus recurrens and parathyroid glands. Currently, anatomical structures can be well identified. Perhaps you could insert a further annotation function in which you can select anatomical structures and these are then annotated with their name. For running through and training operation steps, the model would still need to be significantly expanded with the sequence of OR steps.
(7) Was the navigation in the simulation (menu guidance, settings, etc.) clearly understandable? To what extent does the operation of the simulator fit into your usual clinical workflow (e.g., time expenditure, training)?	Yes, the operation would initially be for training purposes, the time expenditure for operation seems low, a brief instruction is sufficient.	The navigation wasn't quite easy for us, but together we could find our way around. Simpler buttons for table height e.g. would be helpful. Perhaps also clear instructions "pull yourself to the OR table with the left hand" or similar. That's probably but a question of practice. The time expenditure was ok, I had suspected that training would take more time.	The navigation in the simulation seemed quite understandable to me. I consider extra training time for the simulator to be very manageable or not necessary. The simulator would initially be an addition to my usual clinical workflow. This applies but to the majority of my learning effort. If training/simulation of OR steps is possible, then I'm sure that after several attempts it would positively affect the operation time.
(8) Did you experience discomfort, visual irritations, or other stress (e.g., motion sickness) while using the VR glasses or simulator?	No.	No	No.
(9) What additional functions or scenarios would you wish for future versions (e.g., more complex pathologies, more OR steps)? Which realism aspects are particularly decisive for you personally to want to engage with the simulator long-term?	Fat and lymph tissue, an enlarged/nodular thyroid, warnings when too strong luxation of the thyroid exerts stress on the NLR, for long-term engagement, various anatomical scenarios must be possible and ideally also simulation of complications during the operation: stress on the nerve, bleeding, accidental removal of parathyroid gland, as it couldn't be adequately identified (for this, fat tissue is necessary again)	Various levels with different sizes of thyroid/different structures (nodular, soft..), different difficulty levels with nerve course (very thyroid capsule-close versus greater distance etc.), acute bleeding, use of neuromonitoring, realistic OR steps with use of instruments. You could make a game out of it and collect points or e.g. lose when the nerve is severed or get minus points when pulled too strongly on the nerve, that the patient is hoarse after the operation due to reduced mobility of the vocal cord or when it bleeds.	Additional functions: Work with different instruments: e.g. tweezers, Overholt, stick swab, hooks, scissors, bipolar current. Additionally, I would wish for different pathologies: Large/small thyroid, different nerve courses and position of thyroids. Additionally, injuries e.g. of blood vessels should also be possible. Perspectively, I wish for a simulator that enables the course of a complete thyroid operation (incl. possible complications) with best case also feedback regarding precision, errors and injury of anatomical structures.
(10) What did you particularly like about the simulation, and what should be urgently revised in your opinion? Would you like to add anything or do you have further ideas/remarks?	Very real environment that immediately lets you immerse, the surface structures already appear very realistic, anatomically largely correct position, the NLR is realistically very fine, the parathyroid glands very difficult to identify. NV lies too superficially, the missing interstitium/fat tissue, the transition to the skin.	I particularly liked immersing in the OR setting, the possibility to move the thyroid and muscles. As already written above, I would like a game with various levels.	Particularly liked: realistic OR environment. Urgent revision: relation of anatomical structures to each other during deformation.
